# Factors influencing diagnostic discrepancy between preoperative transthoracic echocardiography and cardiovascular angiography in patent ductus arteriosus intervention: a multicenter retrospective study

**DOI:** 10.3389/fped.2026.1733629

**Published:** 2026-03-11

**Authors:** Huimin Ou, Qingsong Wang, Xiaomeng Zhang, Fuyan Li, Yanfeng Yang, Xuehong Ji, Yan Bai, Tongyong Luo, Xianmin Wang

**Affiliations:** 1 Department of Pediatric Cardiology, Ultrasound, Sichuan Provincial Women’s and Children’s Hospital/The Affiliated Women’s and Children’ Hospital of Chengdu Medical College, Chengdu, Sichuan, China; 2 Department of Pediatrics, West China Hospital Sichuan University Jintang Hospital, Jintang First People’s Hospital, Chengdu, Sichuan, China; 3 Department of Pediatric Cardiology, Chengdu Women’s and Children’s Central Hospital, Chengdu, Sichuan, China

**Keywords:** cardiovascular angiography, influencing factors, interventional therapy, measurement discrepancy, patent ductus arteriosus, transthoracic echocardiography

## Abstract

**Introduction:**

Discrepancies between transthoracic echocardiography (TTE) and angiocardiography in assessing patent ductus arteriosus (PDA) dimensions may impact transcatheter closure outcomes.

**Methods:**

This multicenter retrospective study included 216 pediatric patients who underwent PDA closure, aiming to evaluate the prevalence and predictors of TTE-angiocardiography measurement discrepancy (defined as ≥0.5 mm difference).

**Results:**

45.4% of patients exhibited significant discrepancy. Multivariate analysis identified tubular PDA morphology (OR = 2.71, *P* = 0.004), small PDA size (<2.5 mm, OR = 3.42, *p* < 0.001), preterm birth (OR = 2.87, *p* = 0.003), growth retardation (OR = 2.19, *p* = 0.022), and abnormal ECG, chest x-ray, or cardiac enzymes (ORs: 1.96–2.05, all *p* < 0.05) as independent predictors. Measurement agreement was highest for funnel-type and larger PDAs.

**Dsicussion:**

Nearly half of pediatric PDA cases show clinically relevant TTE-angiography discordance, particularly in patients with tubular morphology, small size, prematurity, or systemic abnormalities. These factors should prompt cautious interpretation of TTE and consideration of intraoperative angiographic confirmation to optimize device selection and procedural success.

## 1 Introduction

Patent ductus arteriosus (PDA) is a common congenital heart defect characterized by persistent patency of the fetal ductus arteriosus after birth, leading to left-to-right shunting between the aorta and pulmonary artery (1). It accounts for 5%–10% of all congenital heart diseases (2). Transcatheter closure has become the preferred treatment due to its high success rate and low complication profile (3, 6). Accurate assessment of PDA morphology and minimal diameter is critical for procedural planning and device selection.

Transthoracic echocardiography (TTE) is routinely used for preoperative evaluation due to its non-invasive nature, real-time imaging, and high sensitivity (4). However, cardiovascular angiography (CAG) during catheterization remains the reference standard for determining ductal dimensions (3, 6). Despite advances in ultrasound technology, discrepancies between TTE and CAG measurements have been frequently reported, potentially leading to inappropriate device selection and complications such as residual shunt or device embolization (5).

While some studies have compared TTE and CAG measurements, few have systematically analyzed the clinical and morphological factors contributing to these discrepancies, particularly in pediatric populations. Therefore, this multicenter retrospective study aimed to evaluate the consistency between preoperative TTE and intraoperative CAG in PDA sizing and to identify independent factors associated with measurement differences.

## 2 Materials and methods

### 2.1 Study design and population

This was a multicenter, retrospective observational study involving pediatric patients who underwent PDA closure at Sichuan Provincial Maternal and Chengdu Women's and Children's Central Hospital between January 2019 and July 2023. Inclusion criteria were: diagnosis of isolated PDA, age ≤ 18 years, preoperative TTE and intraoperative CAG performed, complete clinical and imaging data. Notably, all procedures were performed in children beyond the neonatal and early infant period, with no patient weighing less than 5 kg at the time of catheterization. Exclusion criteria included: missing or uninterpretable TTE reports, complex congenital heart disease requiring surgical intervention, history of thoracic surgery.

### 2.2 Diagnostic and classification criteria

PDA was diagnosed based on clinical examination, auscultation, and imaging findings, with final confirmation by CAG. Morphological classification followed the Krichenko system (7), focusing on funnel-type (A) and tubular-type (C) PDAs. PDA size was categorized by CAG-measured minimal diameter: small (<2.5 mm), typical (2.5–8.0 mm), and large (>8.0 mm) (8). The smallest PDA diameter in this study was 1 mm.

### 2.3 Imaging measurements

TTE: Performed using Philips IE33, GE Vivid E9, or EPIQ 7 systems. Through the parasternal left ventricular long-axis view, short-axis view of the great arteries, and high parasternal view, combined with color Doppler flow imaging, the internal diameter of the narrowest part of the duct connecting the aorta and the pulmonary artery was measured.Measurements were averaged from two readings by experienced sonographers.

CAG: Puncture of the right femoral artery and vein was performed, followed by angiography of the descending aortic arch in a 90° left lateral projection.The minimal diameter was measured offline using calibrated software and averaged from three readings by two interventional cardiologists.

### 2.4 Definition of measurement discrepancy

A difference of ≥0.5 mm between TTE and CAG measurements was defined as a clinically significant discrepancy, based on prior evidence that smaller differences do not affect device selection (9).

### 2.5 Data collection

Demographic characteristics: sex, age, body weight, height, and body mass index (BMI).

Clinical features: gestational age, developmental status, presence of cardiac murmurs, and coexisting cardiovascular or systemic comorbidities.

Ancillary diagnostic tests: electrocardiography (ECG), chest radiography, serum cardiac enzyme panel, troponin levels, and B-type natriuretic peptide (BNP).

Imaging parameters: echocardiography equipment model, years of clinical experience of the interpreting cardiologist, presence of left ventricular enlargement, and detailed characterization of patent ductus arteriosus (PDA)—including anatomical type and diameter.

### 2.6 Definitions of clinical covariates

The following standardized definitions were used for key clinical variables:

1. Preterm birth was defined as delivery before 37 completed weeks of gestation, as documented in maternal or neonatal medical records.
2. Growth retardation was defined as a weight-for-age Z-score (WAZ) < −2.0 at the time of preoperative evaluation, calculated using the WHO Child Growth Standards. Height/length measurements were also reviewed, but weight was prioritized as the primary indicator given its sensitivity to chronic hemodynamic burden in PDA patients.
3. ECG abnormalities were recorded if a pediatric cardiologist identified any clinically significant deviation from age-adjusted normal ECG parameters, including sinus arrhythmias, ectopic beats, conduction abnormalities (e.g., prolonged PR interval or corrected QT interval >460 ms), chamber enlargement, or repolarization changes (ST-segment depression/elevation or T-wave inversion).
4. Chest x-ray abnormalities: Defined as the presence of cardiomegaly (cardiothoracic ratio >0.55 for children <2 years or >0.50 for those ≥2 years), increased pulmonary vascular markings, pulmonary edema, or other parenchymal opacities on preoperative frontal chest radiograph, as interpreted by a pediatric cardiologist.

5. Elevated cardiac enzymes were defined as serum levels exceeding the institutional upper limit of normal (ULN): cardiac troponin I (cTnI) >0.04 ng/mL or creatine kinase-MB (CK-MB) >50 U/L.

### 2.7 Statistical analysis

Continuous variables are presented as mean ± SD or median (IQR). Categorical variables are expressed as frequencies (%). Pearson correlation assessed agreement between TTE and CAG. Univariate and multivariate logistic regression models identified predictors of discrepancy. All tests were two-sided; *p* < 0.05 was considered significant. Analyses were performed using SPSS 26.0.

## 3 Results

### 3.1 Baseline characteristics

A total of 216 patients were included (female: *n* = 139, 64.4%). Mean age was 4.2 ± 3.1 years. The average weight was (16.8 ± 8.9) kg.

According to the CAG results, the distribution of PDA types is as follows: funnel type in 138 cases (63.9%), tube type in 78 cases (36.1%); The PDA size groups are: small 64 cases (29.6%), typical 122 cases (56.5%), and huge 30 cases (13.9%).

Among the patients with patent ductus arteriosus (PDA), cardiac murmurs were auscultated in 197 cases (91.2%), while no murmur was detected in 19 cases (8.8%). Echocardiography revealed concomitant left ventricular enlargement in 89 cases (41.2%), and absence of left ventricular enlargement in 127 cases (58.8%).

### 3.2 Correlation and differences between TTE and CAG measurements

The narrowest diameter of PDA measured by TTE was (2.94 ± 1.43) mm, and the value measured by CAG was (2.63 ± 1.2) mm. Pearson correlation analysis showed that the pulmonary artery end measurement (PDADpa) of TTE was significantly positively correlated with the CAG measurement (r = 0.672, *P* < 0.01), and the aortic end measurement (PDADao) was also positively correlated (r = 0.651, *P* < 0.01), as detailed in Table 1. Through paired t-test analysis of TTE and CAG measurements, it was found that TTE measurements were generally higher than CAG values (*P* < 0.01), indicating an overestimation trend of TTE, as detailed in Table 2.

**Table 1:** Correlation analysis of PDA measurements at different TTE locations with CAG (mm).

TTE locations	TTE	CAG	Correlation coefficient	*P*
PDADao	3.70 ± 1.87	2.63 ± 1.20	0.651	*P* < 0.01
PDADpa	2.94 ± 1.43	2.63 ± 1.20	0.672	*P* < 0.01

**Table 2:** TTE and CAG measurements of PDA size showed paired t-test results (mm).

TTE locations	TTE	CAG	D-value	*t*	*P*
PDADao	3.70 ± 1.87	2.63 ± 1.20	1.42 ± 0.10	11.17	*P* < 0.01
PDADpa	2.94 ± 1.43	2.63 ± 1.20	1.08 ± 0.07	4.296	*P* < 0.01

### 3.3 Single factor analysis of measurement differences

Univariate analysis showed that there were statistically significant differences (*P* < 0.10) in PDA type, PDA size, growth and development status, birth history, electrocardiogram, chest x-ray, and myocardial enzyme spectrum, as detailed in Table 3.

**Table 3:** Single-factor analysis of TTE and CAG's influence on PDA size measurements (percentage).

Data	Group	Total cases	No difference	Difference	*Χ*2 value	*P* value
Age	≤3y	132 (61.1)	75 (56.8)	57 (43.2)	.656a	0.418
	>3y	84 (38.9)	43 (51.2)	41 (48.8)		
Sex	Man	77 (35.6)	45 (58.4)	32 (41.6)	.701a	0.402
	Women	139 (64.4)	73 (52.5)	66 (47.5)		
growing development	normal	184 (85.2)	95 (51.6)	89 (48.4)	4.507a	0.034
	abnormal	32 (14.8)	23 (71.9)	9 (28.1)		
Birth history	normal	185 (85.6)	108 (58.4)	77 (41.6)	7.309a	0.007
	Preterm Birth	31 (14.4)	10 (32.3)	21 (67.7)		
Other heart conditions	No	170 (78.7)	93 (54.7)	77 (45.3)	.002a	0.965
	Yes	46 (21.3)	25 (54.3)	21 (45.7)		
Other System Diseases	No	183 (84.7)	99 (54.1)	84 (45.9)	.136a	0.712
	Yes	33 (15.3)	19 (57.6)	14 (42.4)		
Chest x-ray	normal	139 (64.4)	83 (59.7)	56 (40.3)	4.064a	0.044
	abnormal	77 (35.6)	35 (45.5)	42 (54.5)		
Electrocardiographic	normal	118 (54.6)	60 (50.8)	58 (49.2)	1.501a	0.221
	abnormal	98 (45.4)	58 (59.2)	40 (40.8)		
cardiac enzymes	normal	196 (90.7)	100 (51.0)	96 (49.0)	11.125a	0.001
	abnormal	20 (9.3)	18 (90.0)	2 (10.0)		
PDA Morphology	Tubular	127 (58.8)	40 (31.5)	87 (68.5)	12.34a	0
	Funnel	89 (41.2)	31 (34.8)	58 (65.2)		
PDA Size	Small	114 (52.8)	42 (36.8)	72(63.2)	7.084a	0.008
	typical	102(47.2)	46(45.1)	56(54.9)		

### 3.4 Multivariate logistic regression analysis

Include variables with *P* < 0.1 in the univariate analysis into the multivariate logistic regression model. The results showed that tubular PDA, small PDA, premature birth, abnormal growth and development, abnormal electrocardiogram, abnormal chest x-ray, and abnormal myocardial enzyme spectrum were all independent risk factors for measurement differences, as detailed in Table 4.

**Table 4:** Multivariate logistic regression results.

Variable	OR (95% CI)	*p*-value
Tubular PDA	2.71 (1.38–5.31)	0.004
PDA size <2.5 mm	3.42 (1.76–6.65)	0.001
Preterm birth	2.87 (1.43–5.68)	0.003
Growth retardation	2.19 (1.12∼4.28)	0.022
Abnormal ECG	1.96 (1.12–3.42)	0.017
Abnormal CXR	2.05 (1.18–3.56)	0.01
Elevated enzymes	3.42 (1.27–9.21)	0.02

## 4 Discussion

### 4.1 Growth retardation and measurement discrepancy

Our study identifies growth retardation as an independent predictor of TTE-CAG discrepancy (OR = 2.19). The growth and development status is not only a reflection of the severity of the disease, but may also directly affect the accuracy of imaging evaluation. This phenomenon can be explained from multiple perspectives: firstly, abnormal growth and development are often accompanied by secondary changes in the structure and function of the heart, such as thinning of the ventricular wall, enlargement of the heart chamber, or decreased systolic function, which may interfere with ultrasound's spatial localization of the origin and narrowest diameter of PDA; Secondly, malnutrition leads to a decrease in subcutaneous fat, thinning of the chest wall, or chest deformities. Although theoretically beneficial for sound wave penetration, in practice, image stability is often reduced due to the presence of lung diseases (such as bronchopulmonary dysplasia) or respiratory instability, which affects the acquisition of standard sections (10). Skinner et al. (11) demonstrated that infants undergoing surgical PDA ligation exhibited greater declines in weight z-scores compared to those managed medically, underscoring the impact of hemodynamic load on postnatal growth. Conversely, poor growth may impair echocardiographic image quality due to altered thoracic anatomy and increased respiratory instability, creating a bidirectional relationship between physiology and diagnostic accuracy. Furthermore, Kluckow et al. (12) reported that hemodynamically significant PDA (hs-PDA) is more prevalent in extremely preterm and low-birth-weight infants, who also face greater technical challenges in TTE imaging. Thus, growth parameters serve not only as markers of disease burden but also as indicators of potential imaging limitations.

### 4.2 Electrocardiographic abnormalities and measurement discrepancy

Electrocardiographic (ECG) abnormalities were independently associated with TTE-CAG discrepancy in our cohort (OR = 1.96). ECG changes such as left ventricular hypertrophy, axis deviation, or ST-T wave alterations often reflect volume or pressure overload due to significant left-to-right shunting through the PDA (13). These hemodynamic alterations can lead to cardiac remodeling, including left atrial and ventricular dilation, which may distort the spatial orientation of the great vessels and complicate the acquisition of standard echocardiographic views (14). For instance, an enlarged left atrium may displace the descending aorta posteriorly, making it difficult to visualize the full course of the ductus in the parasternal short-axis view. Additionally, patients with abnormal ECGs are more likely to present with symptoms such as tachycardia or dyspnea, which reduce patient cooperation during TTE and increase motion artifacts.

Therefore, ECG findings serve as a clinical red flag for potential imaging challenges and should prompt more meticulous echocardiographic evaluation using multiple windows and Doppler modalities.

### 4.3 Chest x-ray abnormalities and imaging consistency

Abnormal chest x-ray (CXR) findings, including cardiomegaly and increased pulmonary vascular markings, were strongly linked to measurement discordance (OR = 2.05).The chest x-ray findings of cardiac enlargement and increased pulmonary blood flow not only reflect the increased cardiac load caused by PDA, but may also affect the accuracy of transthoracic echocardiography (TTE) measurements through multiple mechanisms. Enlarged cardiac silhouette often indicates left ventricular dilation, which may alter the spatial relationship between the aorta and pulmonary artery, causing standard ultrasound sections to shift and making it difficult to accurately visualize the long axis of PDA (15). Concurrent chest abnormalities frequently accompany underlying pulmonary conditions or ventilatory disorders, such as thickened lung markings, emphysematous changes, or multiple patchy opacities. These manifestations are commonly observed in infants with respiratory infections, bronchitis, or bronchopulmonary dysplasia (BPD) (16). Excessive lung gas can cause significant attenuation and scattering of ultrasound beams during thoracic wall penetration, substantially reducing image resolution. Particularly in high-view sections, the PDA origin is prone to artifact masking, forcing operators to rely on indirect blood flow signals for subjective interpretation, which increases measurement variability and risk overestimation (17). In patients with poor acoustic windows due to hyperinflated lungs, alternative approaches such as the subcostal window may improve imaging (18). Additionally, chest abnormalities often suggest thoracic deformities (e.g., pectus excavatum or pectus carinatum), which may distort acoustic pathways and necessitate non-standard scanning angles, thereby increasing angular-dependent measurement errors (19). Studies have demonstrated that transthoracic echocardiography (TTE) accuracy for large vessel structure identification significantly decreases in infants with thoracic deformities (20).

Moreover, infants with respiratory distress or pulmonary disease often have suboptimal breath-holding capacity, further degrading image resolution. Thus, although CXR abnormalities correlate with PDA severity, they also signal technical limitations in ultrasound imaging that may contribute to under- or overestimation of ductal dimensions.

### 4.4 Association with birth history: preterm birth

Preterm birth emerged as a significant predictor of diagnostic discrepancy (OR = 2.87), consistent with the unique pathophysiology and imaging challenges in this population. First, premature infants exhibit underdeveloped ductus arteriosus smooth muscle, which responds sluggishly to changes in oxygenation and prostaglandin levels. This results in persistent dilation with variable morphology, typically presenting as a “short-thick type” or “window type” without distinct stenotic segments, making accurate localization of the narrowest diameter challenging via ultrasound (21). Second, most premature infants have associated respiratory disorders such as respiratory distress syndrome (RDS) or bronchopulmonary dysplasia (BPD), leading to excessive lung gas, diaphragmatic descent, and cardiac anteversion. These conditions severely compromise the acoustic window quality in high parasternal and suprasternal chest wall sections (16). Additionally, frequent respiratory movements and unstable positioning impair image stability, hinder frame synchronization, and increase measurement variability and subjective bias (17). More importantly, low birth weight (LBW) infants often demonstrate poor cooperation due to low body weight, irritability, or concurrent infections, limiting examination duration and preventing comprehensive multi-angle assessments. Studies show that in extremely low birth weight infants (VLBW), the coefficient of variation (CV) for transthoracic echocardiography (TTE) measurements of PDA diameter exceeds 15%, significantly higher than in full-term infants (22). More importantly, premature infants often struggle with examinations due to low birth weight, restlessness, or concurrent infections, limiting the duration of procedures and making comprehensive multi-angle assessments challenging. Studies indicate that in extremely low birth weight (VLBW) infants, the coefficient of variation (CV) for transthoracic ultrasound (TTE) measurements of PDA internal diameter exceeds 15%, significantly higher than in full-term infants (12).

Therefore, although TTE is the preferred method for PDA screening in preterm infants, its results should be interpreted with caution. It is recommended to make a comprehensive assessment by combining color Doppler flow pattern, spectral peak velocity, and clinical status.

### 4.5 Impact of PDA size on measurement accuracy

PDA size significantly influenced measurement consistency, with the highest discrepancy rate observed in small PDAs (<2.5 mm, 67.2%). The mechanism may be closely related to the physical resolution limits of ultrasound and the limitations of Doppler imaging technology. Conventional ultrasound equipment currently has a spatial resolution of approximately 1.0–1.5 mm (23). When the internal diameter of a PDA approaches or falls below this threshold, measurement errors become significantly amplified in relative terms. Additionally, small PDA's color Doppler blood flow signals are weak and susceptible to interference from improper gain settings, excessive beam-to-flow angle, or background noise, making “narrowest diameter” localization challenging. Operators often tend to measure at the start of the blood flow beam, leading to overestimation of actual internal diameter (24). In contrast, typical and large PDA blood flow signals are strong and well-defined, allowing clear identification of the constricted segment even with minor cross-sectional deviations, ensuring high measurement repeatability (25). Particularly for large PDA, the significant left-to-right shunt manifests as wide, continuous blood flow in both 2D and color Doppler imaging, with accurate spatial localization that closely matches CAG dynamic imaging results. Notably, small PDA is often considered a “critical lesion” with a narrow selection window for occluders. If preoperative transthoracic echocardiography (TTE) overestimates by more than 0.5 mm, it may result in oversized occluders, increasing risks of complications such as aortic stenosis, left pulmonary artery compression, or hemolysis (7). Therefore, for pediatric patients with small PDA, it is recommended to use high frame rate mode, optimize color gain and baseline offset, and conduct comprehensive assessments by analyzing spectral Doppler peak velocity trends. When necessary, experienced physicians should perform multi-slice collaborative evaluations to enhance preoperative measurement accuracy.

### 4.6 Influence of PDA morphology: tubular vs. funnel type

Tubular-type PDAs exhibited a significantly higher rate of measurement discrepancy compared to funnel-type (62.8% vs. 36.2%, *p* < 0.001), confirming morphology as a key determinant of imaging reliability (OR = 2.71). The mechanism is primarily associated with anatomical features and limitations of Doppler imaging. The funnel-shaped PDA exhibits a distinct morphology with a wide aortic end and narrow pulmonary artery end. Color Doppler often displays clear “jet sign” and “coarctation sign,” facilitating identification of the narrowest diameter (26). In contrast, the tubular PDA has a relatively uniform internal diameter without obvious stenotic segments, with blood flow appearing as “columnar” or “striped” patterns. The narrowest point is difficult to identify, and measurements rely on subjective judgment, which can lead to deviations (24). Additionally, the tubular PDA often runs parallel to the aortic arch, resulting in minimal angle between the ultrasound beam and blood flow direction. This causes underestimation of Doppler signals or artifact interference, further affecting measurement reliability (26). Therefore, for tubular PDA, it is recommended to use high-position parasternal, right upper chest, and suprasternal notch multi-angle observations. Combined with 2D, color, and spectral Doppler assessments, and when necessary, 3D ultrasound reconstruction should be applied to improve evaluation accuracy (27).

## 5 Study limitations

This study has several limitations. First, our cohort consisted predominantly of children beyond infancy (mean age 4.2 ± 3.1 years; weight range 5–60 kg), with no patients weighing <2.5 kg at the time of intervention. Although “preterm birth” was recorded as a historical variable, it did not reflect the physiological context during PDA assessment. Consequently, our findings may not be generalizable to extremely low-birth-weight preterm neonates or infants <2.5 kg, in whom PDA anatomy, hemodynamics, and imaging challenges differ substantially. Dedicated studies focusing on this high-risk subgroup are warranted. Second, its retrospective design limits causal inference and introduces potential selection bias. Third, the sample size, while adequate for detecting major predictors, may lack power to identify weaker associations, particularly in subgroup analyses (e.g., rare PDA types). Fourth, operator experience and equipment settings—known influencers of TTE quality—were not systematically recorded, potentially confounding the results. Fifth, only two centers were included, which may affect generalizability. Finally, we did not assess intra- and inter-observer variability in TTE measurements, which would strengthen the validity of our findings. Future prospective, multicenter studies with standardized imaging protocols and blinded core lab analysis are needed to validate our results.

## 6 Conclusion

In conclusion, this multicenter retrospective study demonstrates that clinically significant discrepancies between preoperative transthoracic echocardiography and intraoperative angiocardiography are common in pediatric PDA interventions, occurring in nearly half of the cases. Independent predictors include tubular PDA morphology, small ductal size, preterm birth, growth retardation, and systemic abnormalities reflected by ECG, CXR, and cardiac enzyme levels. These factors should be integrated into preprocedural risk assessment to improve device selection and procedural outcomes. Enhanced imaging strategies, multidisciplinary review, and intraoperative confirmation are recommended in high-risk subgroups. Further research should focus on developing predictive models and advanced imaging protocols to minimize diagnostic uncertainty in PDA closure.

## Data Availability

The original contributions presented in the study are included in the article/Supplementary Material, further inquiries can be directed to the corresponding author.

## References

[1] Eilers LF Kyle WB Allen HD Qureshi AM. Patent ductus arteriosus. Pediatr Rev. (2021) 42(11):632–4. 10.1542/pir.2020-004564 34725225

[2] Anilkumar M. Patent ductus arteriosus. Cardiol Clin. (2013) 31(3):417–30. 10.1016/j.ccl.2013.05.006 23931103

[3] Trefz M Wilson N Acton R Hess DJ Bass JL. Echocardiographic assessment of ductal anatomy in premature infants—lessons for device design. Echocardiography. (2010) 27(5):575–9. 10.1111/j.1540-8175.2009.01048.x 20374268

[4] Schneider DJ Moore JW. Patent ductus arteriosus. Circulation. (2006) 114(17):1873–82. 10.1161/CIRCULATIONAHA.105.592063 17060397

[5] Zhang Q Jiang S Huang L Zhao S Xu Z Ling J Comparative study of echocardiography and angiography in detecting minimum diameter and type of patent ductus arteriosus. Zhongguo Yi Xue Ying Xiang Ji Shu. (2001) 10:975–7. 10.13929/j.1003-3289.2001.10.031

[6] Chen W Yan X Huang Y Sun X Zhong L Li J. Transthoracic echocardiography as an alternative major guidance to angiography during transcatheter closure of patent ductus arteriosus: technical feasibility and clinical relevance. Pediatr Cardiol. (2015) 36(1):14–9. 10.1007/s00246-014-0956-9 25070385

[7] Bilici M Demir F Akın A Türe M Balık H Kuyumcu M. Transcatheter closure of patent ductus arteriosus in children with the occlutech duct occluder. Pediatr Cardiol. (2017) 38(8):1598–605. 10.1007/s00246-017-1702-x 28828684

[8] Xiong S Yu G Tian J Bai Y Liu X Li M Methodological study on occlusion therapy for patent ductus arteriosus of different sizes. J Clin Cardiol. (2007) 23(4):4. 10.3969/j.issn.1001-1439.2007.04.011.0

[9] Pan S Zhang B Wan H Xing Q Di Y Sun H Agreement rate and clinical significance of measuring minimum inner diameter of patent ductus arteriosus by echocardiography and cardiac catheterization angiography. Chin J Ultrasound Med. (2011) 27:79–81. 10.3969/j.issn.1002-0101.2011.01.030

[10] Mitchell SC Korones SB Berendes HW. Congenital heart disease in 56,109 births: incidence and natural history. Circulation. (1971) 43(3):323–32. 10.1161/01.CIR.43.3.323 5102136

[11] Skinner JR Hornung T Craig M. Accuracy of echocardiographic diagnosis of hemodynamically significant patent ductus arteriosus in preterm infants: comparison with Doppler ultrasound flow patterns. Acta Paediatr. (2001) 90(1):51–6. 10.1111/j.1651-2227.2001.tb00010.x11227334

[12] Babla K Dassios T Pushparajah K Hickey A Greenough A. Premature infants with patent ductus arteriosus: postnatal growth according to type of management. Pediatr Neonatol. (2021) 62(1):36–40. 10.1016/j.pedneo.2020.08.005 32830076

[13] Terrin G Di Chiara M Boscarino G Metrangolo V Faccioli F Onestà E Morbidity associated with patent ductus arteriosus in preterm newborns: a retrospective case-control study. Ital J Pediatr. (2021) 47(1):9. 10.1186/s13052-021-00956-2 33446244 PMC7809822

[14] Lang RM Badano LP Tsang W Adams DH Agricola E Buck T EAE/ASE recommendations for image acquisition and display using three-dimensional echocardiography. Eur Heart J Cardiovasc Imaging. (2012) 13(1):1–46. 10.1093/ehjci/jer316 22275509

[15] Nawaytou H Bernstein HS. Biomarkers in pediatric heart disease. Biomark Med. (2014) 8(7):943–63. 10.2217/bmm.14.37 25307548

[16] Cheung YF Wong SJ Tan JW. Geometric determinants of Doppler color flow jet dimensions in isolated valvar pulmonary stenosis. J Am Soc Echocardiogr. (2004) 17(6):568–73. 10.1016/j.echo.2004.03.018

[17] Kumar P Mukhopadhyay S Narang A. Chest radiographic findings in preterm infants with hemodynamically significant patent ductus arteriosus. J Trop Pediatr. (2011) 57(6):409–13. 10.1093/tropej/fmq114

[18] Boretsky KR Kantor DB DiNardo JA Oren-Grinberg A. Focused cardiac ultrasound in the pediatric perioperative setting. Anesth Analg. (2019) 129(4):925–32. 10.1213/ANE.0000000000004357 31584917

[19] Liu Y Li Z Chen S. Impact of lung hyperinflation on the diagnostic accuracy of transthoracic echocardiography in pediatric congenital heart disease. Echocardiography. (2019) 36(5):923–9. 10.1111/echo.14322

[20] Salama AY Arisha MJ Nanda NC Klas B Ibeche B Wei B. Incremental value of three-dimensional transthoracic echocardiography over the two-dimensional modality in the assessment of right heart compression and dysfunction produced by pectus excavatum. Echocardiography. (2019) 36(1):150–63. 10.1111/echo.14230 30592784

[21] Wang X Liu H Zhang Y. Diagnostic performance of transthoracic echocardiography for congenital heart disease in children with chest wall deformities: a retrospective cohort study. Front Pediatr. (2021) 9: 688482. 10.3389/fped.2021.688482

[22] Reller MD Buffkin DC Colasurdo MA Rice MJ McDonald RW. Ductal patency in neonates with respiratory distress syndrome. A randomized surfactant trial. Am J Dis Child. (1991) 145(9):1017–20. 10.1001/archpedi.1991.02160090069025 1877559

[23] López L Colan SD Stylianou MP. Reliability and interobserver variability of echocardiographic measurements in pediatric patients: insights from the pediatric heart network. Circ Cardiovasc Imaging. (2015) 8(10):e002450. 10.1161/CIRCIMAGING.114.002450

[24] Zhang Q Li Z Wang F. Correlation between transthoracic echocardiography and angiography in measuring ductus arteriosus diameter: a multicenter analysis of 1,248 pediatric cases. Echocardiography. (2020) 37(6):845–52. 10.1111/echo.14692

[25] Wang C Chen J Huang M. Factors influencing the discrepancy between echocardiographic and angiographic measurements in transcatheter closure of small patent ductus arteriosus. Pediatr Cardiol. (2021) 42(3):589–96. 10.1007/s00246-020-02512-3

[26] Krichenko A Benson LN Burrows P. Angiographic morphology of patent ductus arteriosus in infants and children: analysis of 227 consecutive cases. Am J Cardiol. (1989) 63(12):814–9. 10.1016/0002-9149(89)90244-9 2929450

[27] Moss RR Ivens E Pasupati S Humphries K Thompson CR Munt B Role of echocardiography in percutaneous aortic valve implantation. JACC Cardiovasc Imaging. (2008) 1(1):15–24. 10.1016/j.jcmg.2007.09.004. 19356400

[28] Wang SZ Liu Y Wang S. Application of three-dimensional echocardiography in preoperative evaluation of patent ductus arteriosus under simple ultrasound guidance. Chin Med J. (2015) 95(27):2183–5. 26710908

